# Stop spoon dosing: milliliter instructions reduce inclination to spoon dosing

**DOI:** 10.1186/s13104-015-1809-1

**Published:** 2016-01-21

**Authors:** Koert van Ittersum, Brian Wansink

**Affiliations:** Faculty of Economics and Business, University of Groningen, Nettelbosje 2, 9747 AE Groningen, The Netherlands; Cornell University, 15 Warren Hall Ithaca, Ithaca, NY 14853-7801 USA

**Keywords:** Liquid medicine, Dosage errors, Spoon dosing, Milliliters, Medicine labels

## Abstract

**Background:**

Does the use of teaspoon units in dose recommendations on Drug Facts panels of liquid medicine lead to dosing errors and could any such errors be reduced if millimeter units were used instead?

**Findings:**

Participants given dosing instructions in teaspoon units were twice as likely to choose a kitchen teaspoon as those given instructions in milliliter units (31.3 vs. 15.4 %).

**Conclusion:**

Our results suggest that spoon usage—and the inherent risk of dosage errors—could be reduced by more than 50 % simply by changing the units of measurement given in dosing instructions.

## Findings

Medicine dosage instructions for children are often irresponsible. Over 86 % of all over-the-counter drugs for children and adolescents do not give responsible dosage guidance according to the US Food and Drug Administration (FDA) [1]. This is of greater concern with liquid medicines, since their dosing devices are a major cause of dosing errors and pediatric poisonings [2–4]. For instance, using teaspoons leads to under-serving by 8.4 % and using tablespoons leads to over-serving by 11.6 % [5]. Despite converging evidence, the pharmaceutical industry continues to use teaspoon units in dose recommendations on Drug Facts panels, creating a false assumption that spoons are the preferred dosing device [5]. This study examines the extent to which teaspoon units in dosage guidance increases patient risk of dosing errors compared to a less ambiguous and less estimable measurement, such as milliliters.

## Methods

In conducting this research, data were collected with approval from both Georgia Institute of Technology (the former employer of the first author) and Cornell University’s Institutional Review Boards. The participants were part of an IRB approved subject pool at Georgia Tech that involved approximately 200–225 students. Being a member of the subject pool, participants were informed via email about upcoming studies, including this one.

Upon entering the lab, people did receive a consent form with study information.

The last sentence of the consent form read “By answering the survey questions you have agreed to be a participant in this study”. To determine if teaspoon units in dose recommendations bias patient choice of measuring device for dispensing over-the-counter medicine, 194 university students (118 male; mean age, 20.6 years [SD, 1.5]) participated in a for-credit, between-subject design study. All participants completed a consent form to participate in this research.

Participants were randomly assigned to one of three conditions where dose recommendations for cough medicine were given in either (1) teaspoons (1 teaspoonful every 12 h), (2) milliliters (5 mL every 12 h), or (3) both teaspoons and milliliters (1 teaspoonful (5 mL) every 12 h). All three dose recommendations stated: “The FDA recommends against the use of kitchenware to medicate liquid medicines.” Next, all participants were shown pictures of four different dosing devices: (1) a kitchen teaspoon, (2) a measuring cup with milliliter (mL) marks, (3) a measuring cup with teaspoon (tsp) marks, and (4) a measuring cup with both milliliter (mL) and teaspoon (tsp) marks. Participants were then asked which device they would use.

## Results

Participants’ choice of dosing device was significantly influenced by the units of measurement in the instructions (χ^2^(6) = 96.5, *P* < .001). As the Table 1 indicates, participants who were given dosing instructions in teaspoon units were twice as likely to choose a kitchen teaspoon as those given instructions in milliliter units (31.3 vs. 15.4 %). Those given joint teaspoon/milliliter units were still 1.5 times more likely to select a kitchen teaspoon (23.1 vs. 15.4 %).

**Table 1:** Units of measurement on Drug Facts panel bias selection of dosing device

Selected dosing device	Units of measurement on Drug Facts panel		
	In teaspoons (%)	In milliliters (%)	In teaspoons & milliliters (%)
Teaspoon	31.3	15.4	23.1
Measuring cup (tsp)	59.4	1.5	46.2
Measuring cup (mL)	0.0	56.9	9.2
Measuring cup (tsp/mL)	9.4	26.2	21.5
	100	100	100

In this study, 61 of 177 participants (34.5 %) reported using kitchen spoons most frequently when dosing liquid medicines at home. Of the habitual spoon-users who received dose recommendations in teaspoons, 60.9 % chose a teaspoon and none of them chose a milliliter measuring cup. However, among spoon-users given dose recommendations in milliliters, the teaspoon and the milliliter measuring cup were equally popular, both being selected by 48.3 %.

## Discussion

Spoons are commonly used to measure medicine for both adults and for children in their care, and their estimated use ranges from as high as 70 %, [5] to this study’s more conservative estimate of one in three people. This range is significant and the results of this study show that this overreliance on spoons—and the risk of dosage errors—could be reduced by more than 50 % by simply changing the units of measurement given in dosing instructions from teaspoons to milliliters. It is important to note, however, that using milliliters may not be the only solution. Other dosing devices—such as oral medicine syringes and medicine droppers—could also be effective and should be tested on a more heterogeneous population sample.

In light of these results, the authors call upon the FDA and the pharmaceutical industry to stop using teaspoon units in dose recommendations. In the interim, and consistent with recommendations by safety organizations like the Institute for Safe Medication Practices (http://www.ISMP.org), doctors and pharmacists can advise patients and especially, caregivers of children, to use more accurate dosing devices such as cups, droppers and syringes.
