# Erratum to: Stop spoon dosing: milliliter instructions reduce inclination to spoon dosing

**DOI:** 10.1186/s13104-016-1929-2

**Published:** 2016-03-14

**Authors:** Koert van Ittersum, Brian Wansink

**Affiliations:** Faculty of Economics and Business, University of Groningen, Nettelbosje 2, 9747 AE Groningen, The Netherlands; Cornell University, 15 Warren Hall, Ithaca, NY 14853-7801 USA

## Erratum to: BMC Res Notes (2016) 9:33 DOI 10.1186/s13104-015-1809-1

Unfortunately, the original version of this article [1] contained an error. The following sentence was incorrectly included in the Methods section, “All participants completed a consent form to participate in this research.”

The third paragraph in the Methods section should have read, ‘The last sentence of the consent form read “By answering the survey questions you have agreed to be a participant in this study”. To determine if teaspoon units in dose recommendations bias patient choice of measuring device for dispensing over-the-counter medicine, 194 university students (118 male; mean age, 20.6 years [SD, 1.5]) participated in a for-credit, between-subject design study.'
